# Re-evaluation of the Haarlem *Archaeopteryx* and the radiation of maniraptoran theropod dinosaurs

**DOI:** 10.1186/s12862-017-1076-y

**Published:** 2017-12-02

**Authors:** Christian Foth, Oliver W. M. Rauhut

**Affiliations:** 1 Department of Geosciences, Université de Fribourg, Chemin du Musée 6, 1700 Fribourg, Switzerland; 2 SNSB-Bayerische Staatssammlung für Paläontologie und Geologie, Ludwig-Maximilians-University Munich, Department for Earth and Environmental Sciences, and GeoBioCenter, Richard-Wagner-Str. 10, 80333 Munich, Germany; 3 Current address: Staatliches Museum für Naturkunde Stuttgart, Rosenstein 1, 70191 Stuttgart, Germany

**Keywords:** Maniraptora, Anchiornithidae, Late Jurassic, Biogeography, Radiation

## Abstract

**Background:**

*Archaeopteryx* is an iconic fossil that has long been pivotal for our understanding of the origin of birds. Remains of this important taxon have only been found in the Late Jurassic lithographic limestones of Bavaria, Germany. Twelve skeletal specimens are reported so far. *Archaeopteryx* was long the only pre-Cretaceous paravian theropod known, but recent discoveries from the Tiaojishan Formation, China, yielded a remarkable diversity of this clade, including the possibly oldest and most basal known clade of avialan, here named Anchiornithidae. However, *Archaeopteryx* remains the only Jurassic paravian theropod based on diagnostic material reported outside China.

**Results:**

Re-examination of the incomplete Haarlem *Archaeopteryx* specimen did not find any diagnostic features of this genus. In contrast, the specimen markedly differs in proportions from other *Archaeopteryx* specimens and shares two distinct characters with anchiornithids. Phylogenetic analysis confirms it as the first anchiornithid recorded outside the Tiaojushan Formation of China, for which the new generic name *Ostromia* is proposed here.

**Conclusions:**

In combination with a biogeographic analysis of coelurosaurian theropods and palaeogeographic and stratigraphic data, our results indicate an explosive radiation of maniraptoran coelurosaurs probably in isolation in eastern Asia in the late Middle Jurassic and a rapid, at least Laurasian dispersal of the different subclades in the Late Jurassic. Small body size and, possibly, a multiple origin of flight capabilities enhanced dispersal capabilities of paravian theropods and might thus have been crucial for their evolutionary success.

**Electronic supplementary material:**

The online version of this article (10.1186/s12862-017-1076-y) contains supplementary material, which is available to authorized users.

## Background

Having been discovered only 2 years after Darwin’s [1] prediction that intermediate forms of different clades should be present in the fossil record, the famous ‘Urvogel‘ *Archaeopteryx* from the Solnhofen limestones of southern Germany [2] has not only played an important role in the initial discussion of Darwin’s theory [3], but became the ‘yard-stick’ for bird evolution – the taxon to which any new relevant discovery is compared [4, 5]. However, *Archaeopteryx* remained the only Jurassic paravian theropod known from diagnostic material for more than 100 years.

Recent discoveries in the probably Oxfordian Tiaojishan Formation [6, 7] of eastern China have changed this picture. In the past 15 years, numerous taxa of maniraptoran coelurosaurs were found in this formation. Although the phylogenetic relationships of these taxa are still disputed [4, 5, 8, 9], at least the genera *Anchiornis* and *Xiaotingia* are usually considered to form a monophyletic, endemic clade [4, 8–10], which were recently considered to be the oldest known avialans [9]. Indeed, with the exception of some fragmentary and questionable remains (mainly isolated teeth), no paravian theropod but *Archaeopteryx* has so far been reported from Jurassic rocks outside China.

**Fig. 1:**
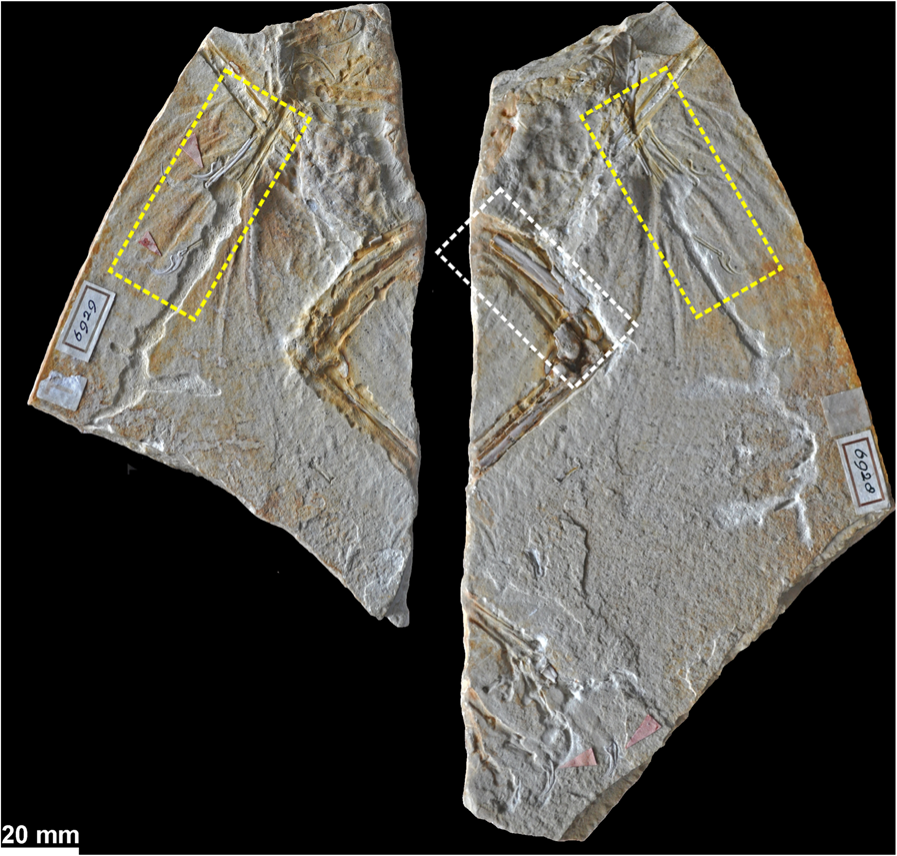
Overview of the “Haarlem specimen”, holotype of *Ostromia crassipes* (Meyer, 1857). Counterslab, Teylers Museum TM 6929 (*left*) and main slab, TM 6928 (*right*). Rectangles indicate areas detailed in Fig. 3 (*yellow*) and Fig. 6 (*white*)

Although the discovery of the first *Archaeopteryx* skeleton was announced by von Meyer [2], a fragmentary paravian theropod was found 6 years earlier, but misidentified as a new species of the pterosaur *Pterodactylus*, *P. crassipes* [11]. It was not until 1970 that this specimen (Fig. 1) was correctly identified as theropod and referred to the genus *Archaeopteryx* [12], and it has since been known as the Haarlem specimen, as it is kept in Teylers Museum in Haarlem, the Netherlands [13].

A re-examination of the Haarlem specimen of *Archaeopteryx* failed to identify any shared apomorphic/diagnostic characters with other *Archaeopteryx* specimens, but revealed important differences that make a referral to this taxon untenable, but indicate a relationship to *Anchiornis* and relatives.

## Methods

### Anatomical comparison

Both the original of the Haarlem specimen, as well as a high quality cast kept at the Bayerische Staatssammlung für Paläontologie und Geologie in Munich were studied in detail, using binokular microscopes. The specimen was compared to other specimens of *Archaeopteryx* on the basis of personal observations of one or both of the authors on all 12 known specimens of *Archaeopteryx*, except the London and Maxberg specimens. However, of these specimens, high quality casts held at the BSPG were studied. Specimens of *Anchiornis*, including the holotype, and several other theropods of the Jehol Biota were personally studied by one of us (CF), and high-resolution photographs of additional specimens of *Anchiornis*, *Eosinopteryx*, and *Aurornis* were graciously provided by Markus Moser, Pei Rui, Helmut Tischlinger, and Wang Xiaoli.

The incomplete preservation of the Haarlem specimen (Fig. 1) precludes exact measurements for most long bones, which is most probably the reason why this specimen has not been included in previous evaluations of the proportions of *Archaeopteryx* [14, 15]. Ostrom [16] and Wellnhofer [13] provided measurements for this specimen, but noted that most of them where either incomplete, or based on estimations. In order to compare the Haarlem specimen to other *Archaeopteryx* specimens, we thus took new measurements from a high quality cast held at the Bayerische Staatssammlung für Paläontologie und Geologie (SNSB-BSPG 1971 I 211), and verified anatomical features by personal observations of the original specimen (Teylers Museum TM 6928, 6929), which was examined in July 2016.

Only elements that could be measured directly or estimated with high certainty were considered here (Table 1). Elements that can be measured with precision include metacarpals I and III, manual phalanx I-1, manual unguals I and III, as well as pedal ungual III. Parts of the metacarpals or their impressions are found on both the main and counterslab and can be used to establish the length of these elements. Thus, the proximal end of metacarpal I is preserved on the main slab, whereas an impression of its distal end is present on the counterslab. The proximal part of metacarpal II is clearly preserved as impression on the counterslab, but the distal end of this element is obscured by a dissolved area, a typical feature of the limestones of Jachenhausen [17]. However, as the metacarpus was obviously preserved in complete articulation, the proximal end of metacarpal II is a good indicator for the approximate position of the proximal end of metacarpal III, and most of the shaft and the distal end of the latter are preserved as impression on the counterslab. Most of manual phalanx I-1 is preserved on the counterslab, and both its proximal and distal extremities are clearly marked as sharp impressions so that the length of this bone can be established with certainty. Two manual unguals are preserved. One of them is associated, though not articulated with phalanx I-1 and is therefore interpreted as the ungual of digit I. The other ungual is articulated with the distal end of a slender phalanx distal to the dissolved area on the counterslab. As the phalanx seems to be too slender to represent the much more robust digit II, we concur with Ostrom [16] and Wellnhofer [13] that this element represents the ungual of digit III. The length of the unguals was measured perpendicular to a line drawn through the dorsal and ventral extremity of the proximal articular end [18]. Two pedal unguals and several impressions are present on the main slab. One of these unguals can be measured precisely and is clearly associated with the impressions of the third digit of the right foot.

**Table 1:** Selected measurements of paravian theropods

Specimen	mcI	mcIII	mpI-1	muI	muIII	tibia	mt	puIII
London	–	–	–	–	–	80.7	44.1	10.3
Berlin	8.3	24.8	20.5	9.6	7.3	68.5	37	8.1
Maxberg	9.8	30	25	9.9	8.4	76.5	41.5	–
Eichstätt	5.5	16.5	15.6	6.9	5.2	52.5	30.2	6.1
Solnhofen	–	–	28	13.8	10.4	92	47.5	11.7
München	7.2	23.5	20	9.2	6.7	73	40	7.4
Thermopolis	6.6	22	19.5	–	–	74,6	39.6	–
11th	9.9	31.5	23.3	11.6	10	76.3	40.3	8.1
Haarlem	10.5	23.2	23.1	9.5	8.3	80	48	7.8
*Anchiornis*	12.5	30.5	26.2	15.6	13.8	106.4	55.2	13.7

Selected measurements (in mm) of the Haarlem specimen and several other specimens of *Archaeopteryx*, as well as the anchiornithid *Anchiornis*. Measurements of the Haarlem specimen and measurements of unguals (except for *Anchiornis*) were taken by us, other measurements taken from Wellnhofer [13] and Hu et al. [32]. Abbreviations: mp, manual phalange; mc, metacarpal; mt, metatarsus; mu, manual ungual; pu, pedal ungual

The estimated elements are the tibiotarsus and metatarsus. As noted by Ostrom [16] and Wellnhofer [13], the preservation of the femora, tibia and fibulae, together with the position of the remains of the feet indicate that the legs were preserved in complete articulation. Thus, although the distal end of the tibiotarsus and the proximal end of the metatarsus are missing, their approximate lengths can be estimated with a probably relatively small margin of error based on where these elements would intersect. Our estimations are in accordance with those given by Ostrom [16], but slightly higher than those provided by Wellnhofer [13]. The reason for this seems to be that Wellnhofer seems to have measured the tibiotarsus of the right leg, which is slightly less flexed than the left leg and thus displaced distally in respect to the latter, whereas the impression of the metatarsus is of the left element.

In order to statistically evaluate the significance of differences in proportions, we performed one sample t- and z-tests for the ratios of metacarpal III / metacarpal I, manual phalanx I-1 / metacarpal I, manual phalanx I-1 / metacarpal III, manual ungual I / metacarpal I, manual ungual III / metacarpal III and tibiotarsus / metatarsus, testing the Haarlem specimen against those specimens that can be classified as *Archaeopteryx* (or at least a monophyletic Archaeopterygidae) based on the diagnosis provided by Foth et al. [9]. The one sample tests compare the value in question with the range of the comparative statistical population, in this case up to seven other specimens of *Archaeopteryx*, under the assumption of a normal distribution, to evaluate the probability that this value represents the same population, based on probability tables that account for sample size. Given the small sample size, the statistical power of these tests is rather low. For the t- and z-tests, the values of the Maxberg specimen have not been included; although the observable morphology and proportions of this specimen are consistent with an identification as *Archaeopteryx*, none of the diagnostic characters of this taxon are preserved in this specimen, so that this identification should be regarded as tentative.

### Phylogenetic and paleogeographic analyses

In order to test the affinities of the Haarlem specimen, we added this specimen to an updated version of the phylogenetic matrix of Foth et al. [9]. Several character codings were revised, and one additional character was added. Character coding for the OTU *Archaeopteryx* was based on the pooled information from all specimens that can be identified as belonging to a monophyletic clade (here regarded as the genus *Archaeotperyx*, regardless of alternative generic names proposed for several specimens and the still unresolved species taxonomy) based on the diagnostic characters proposed by Foth et al. [9] and additional unpublished information gathered by the authors. Thus, for our analysis, this genus currently includes the London, Berlin, Eichstätt, Solnhofen, Munich, Daiting, Thermoplois, 11th, and 12th specimens, and, most probably, also the Maxberg specimen. The resulting data matrix thus contained 132 OTUs and 561 characters, and is deposited on morphobank (www.morphobank.org) under project 2532. The matrix was analyzed using equally weighted parsimony in TNT [19] using a heuristic search with 1000 replicates of Wagner trees followed by TBR branch swapping (holding 10 trees per replicate). To evaluate node support 1000 bootstrap replicates were calculated.

To establish the ancestral range of maniraptorans, we used a Statistical Dispersal-Vicariance Analysis (S-DIVA) [20], using the software RASP [21]. For the S-DIVA analysis, we ran a second phylogenetic analysis in TNT with only 100 replicates, to obtain a more manageable tree file. The analysis resulted in 190 equally parsimonious trees, the strict consensus of which is identical to that recovered in the more extensive analysis described above. These 190 trees were fed into RASP and used to calculate a condensed tree (=majority rule consensus) in this program for the biogeographic analysis.

One problem for biogeographic analyses based on such a large dataset is the stratigraphic range of the taxa covered [22]. Indeed, the phylogenetic matrix includes taxa ranging from the Middle Jurassic (*Proceratosaurus*: Bathonian) to Recent (several modern genera of birds) and thus covers roughly 167 million years. This time span saw profound changes in geography, with the Middle Jurassic representing the ultimate stages of the supercontinent Pangea, its initial break-up in the Late Jurassic and its fragmentation during the Cretaceous, up to the configuration of the modern continents during the Cenozoic. Thus, the current paleogeographical regions might have only limited value for analyzing biogeographic patterns at the time implicated for the major radiation of maniraptorans (Middle Jurassic). However, as the vast majority of taxa come from a rather limited number of relatively small regions, including eastern Asia (China and Mongolia), Europe, western North America, Madagascar, and southern South America (Argentinean Patagonia), these areas can be used as proxies for the geographic distribution of these animals over the entire time span. To further test the influence of long time spans and thus also possible changes in the geographic distribution of taxa used as OTUs as opposed to their possibly much more antique ancestors, we reran two additional time-sliced analyses [22, 23], one with only Jurassic taxa and their interrelationships included, and one including Jurassic and Early Cretaceous taxa. The second analysis was performed because Jurassic taxa are actually poorly represented in the data set, as few Jurassic coelurosaurs have been described so far (only 16 of 132 taxa of the complete data set represent this time period, including the two outgroup taxa).

## Results

### Differences in proportions

**Fig. 2:**
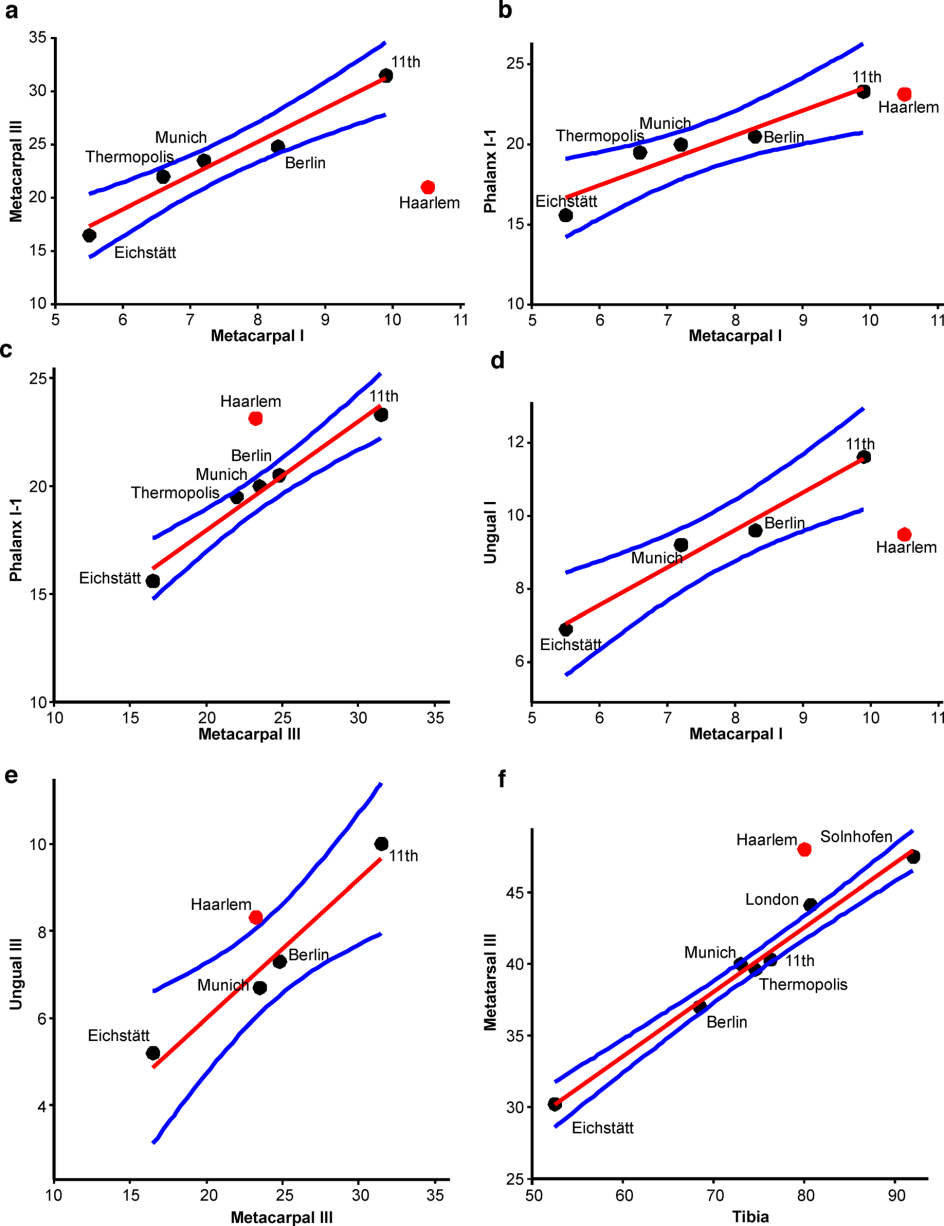
Scatterplots for several proportions that can be established in *Ostromia* (red dots), comparing this taxon with specimens that can certainly be referred to *Archaeopteryx* (black dots). Red line shows the ordinary least squares regression fit and blue lines represent the 95% confidence intervals for the distribution of values of *Archaeopteryx*. **a** Ratio between metacarpal III and metacarpal I. **b** Ratio between phalanx I-1 and metacarpal I. **c** Ratio between phalanx I-1 and metacarpal III. **d** Ratio between ungual I and metacarpal I. **e** Ratio between ungual III and metacarpal III. **f** Ration between metatarsal III and tibia

Due to its incompleteness, none of the diagnostic characters for the genus *Archaeopteryx* listed by Foth et al. [9] is preserved in the Haarlem specimen, so that a direct evaluation of the taxonomic referral is not possible. However, the preserved elements show several differences to the corresponding parts of those specimens that can securely be referred to *Archaeopteryx*. Despite the limited data, several significant differences in proportions between the Haarlem specimen and other specimens of *Archaeopteryx* are obvious (Fig. 2; Table 2). Thus, the ratio between the length of metacarpal III and metacarpal I (Fig. 2a) varies between 2.99 (Berlin specimen) and 3.33 (Thermopolis specimen) in *Archaeopteryx*, but is 2.21 in the Haarlem specimen. Likewise, the ratio between manual ungual I and metacarpal I (Fig. 2b) varies between 1.16 (Berlin specimen) and 1.28 (Munich specimen) in *Archaeopteryx*, but is 0.90 in the Haarlem specimen. A possible explanation for this difference could be that the ungual has been wrongly identified and might represent the ungual of digit II or even of digit III of the left manus. However, ungual II does not differ considerably in size from the ungual of the first digit in other specimens of *Archaeopteryx* [13], and the difference in size between this ungual and the preserved ungual of the right digit III, as well as the close association of this ungual with phalanx I-1 argue against this interpretation. A further value in which the Haarlem specimen differs significantly from other specimens of *Archaeopteryx* is the length of the tibiotarsus in relation to the metatarsus (Fig. 2f), which varies between 1.74 (Eichstätt specimen) and 1.94 (Solnhofen specimen) in *Archaeopteryx*, but is 1.67 in the Haarlem specimen. As the length of the tibiotarsus and metatarsus can only be estimated in the Haarlem specimen, this difference should, however, be seen with caution. Ostrom [16] and Wellnhofer [13] noted the unusually long metatarsus in the Haarlem specimen as well, but as the distal end of the tibiotarsus and the proximal end of the metatarsus are missing, they speculated that this unusual measurement was due to a displacement of the metatarsus from the ankle. However, as both knee joints are preserved in articulation, with hardly any space in between the femur and the tibia, there is no reason to assume a displacement of the metatarsus by as much as 5 mm, the value needed to fall within the extremes of the range exhibited by the specimens that can securely be referred to *Archaeopteryx*. Thus, although the exact value might differ slightly, we tentatively regard this difference in proportions as real.

**Table 2:** Skeletal proportions in *Archaeopteryx* and *Anchiornis*

	mcIII/ mcI	mpI-1/ mcI	mpI-1/ mcIII	muI/ mcI	muIII/ mcIII	tibia/ mt
London	–	–	–	–	–	1.83
Berlin	2.99	2.47	0.83	1.16	0.29	1.85
Eichstätt	3.00	2.84	0.95	1.25	0.32	1.74
Solnhofen	–	–	–	–	–	1.94
München	3.26	2.78	0.85	1.28	0.29	1.83
Thermopolis	3.33	2.95	0.89	–	–	1.88
11th	3.18	2.35	0.74	1.17	0.32	1.89
*Anchiornis*	2.46	2.11	0.86	1.22	0.45	1.93
Haarlem	2.21	2.20	1.00	0.90	0.36	1.67
*t*-value	13.603	4.195	4.287	10.354	6.916	7.716
*p*-value	<0.001	<0.020	<0.020	<0.002	<0.01	<0.001
*z*-value	6.083	1.876	1.917	5.179	3.458	2.916
*p*-value	<0.001	0.038	0.024	<0.001	<0.001	0.001

Ratio of skeletal elements in the Haarlem and other *Archaeopteryx* specimens (which can be diagnosed) and statistical differences (*t*-test and *z*-test) between the Haarlem specimen and *Archaeopteryx*. Abbreviations: mp, manual phalange; mc, metacarpal; mt, metatarsus; mu, manual ungual

Both statistical tests rejected the null hypothesis that the ratios for the Haarlem specimen were taken from the same population as the ratios for the securely identified *Archaeopteryx* specimens at *p* < 0.002. In addition, the Haarlem specimens differs from *Archaeopteryx* in the ratio between the manual phalanx I-1 and metacarpal 1, manual phalanx I-1 and metacarpal III and manual ungual III and metacarpal III (Table 2). Although the statistical power of the tests is low, given the low sample size, these differences indicate that there is little reason to refer this specimen to *Archaeopteryx*.

### Osteological differences

**Fig. 3:**
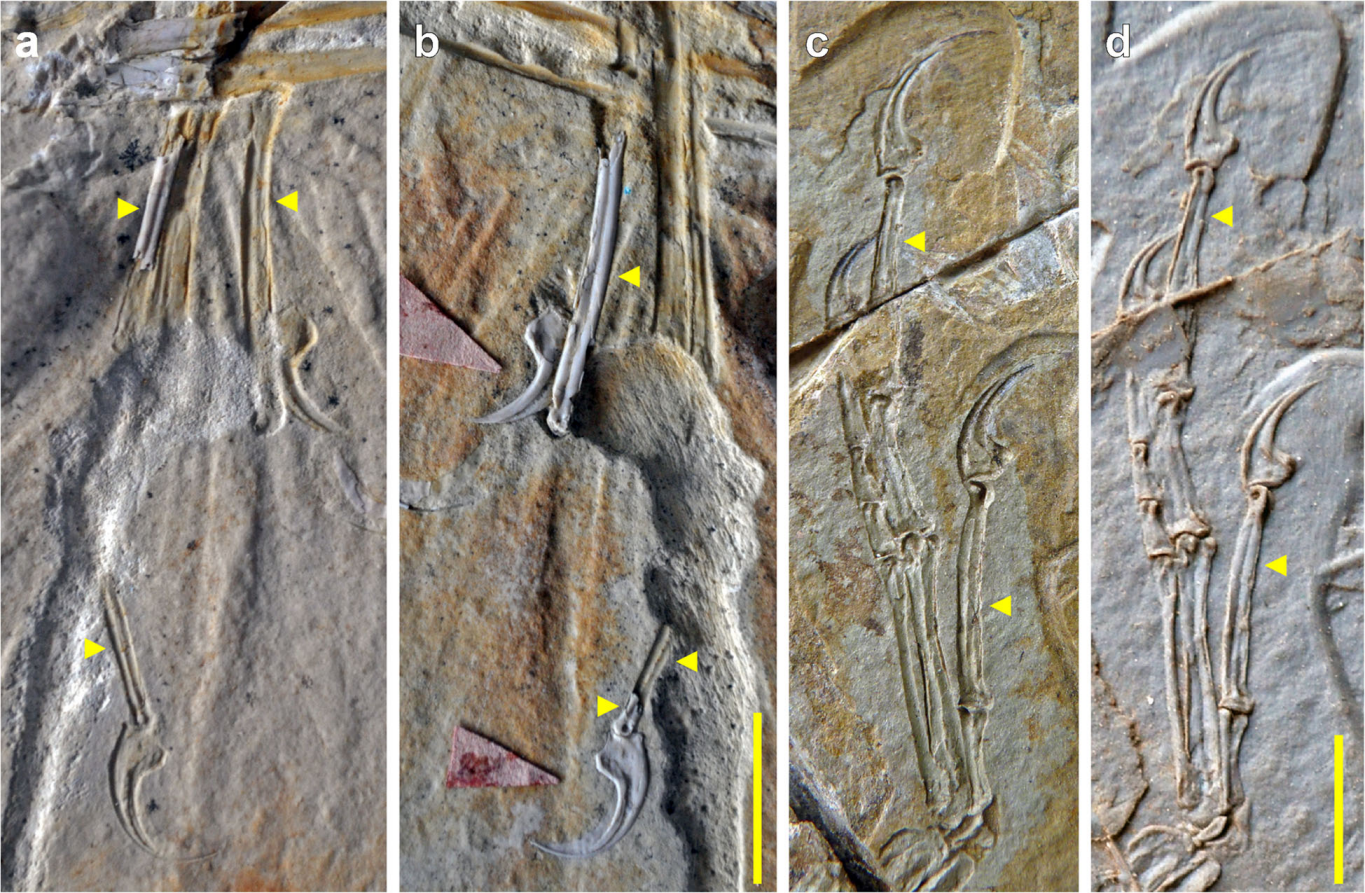
Anatomical details of the manus of *Ostromia crassipes* and *Anchiornis huxleyi*. **a** Detail of the preserved elements of the right manus on the main slab, showing longitudinal furrows (or their impressions) in metacarpal III and the manual phalanges (arrows). **b** Detail of the preserved elements of the right manus on the counterslab of the holotype of *Ostromia crassipes* (TM 6929), showing longitudinal furrows (or their impressions) in the manual phalanges (arrows). **c, d** Impression (**c**) and high-resolution cast (**d**) of the left manus of the holotype of *Anchiornis huxleyi* (Institute of Vertebrate Paleontology and Paleoanthropology IVPP V 14378), showing longitudinal furrows in the manual phalanges (arrows). All scale bars are 10 mm

**Fig. 4:**
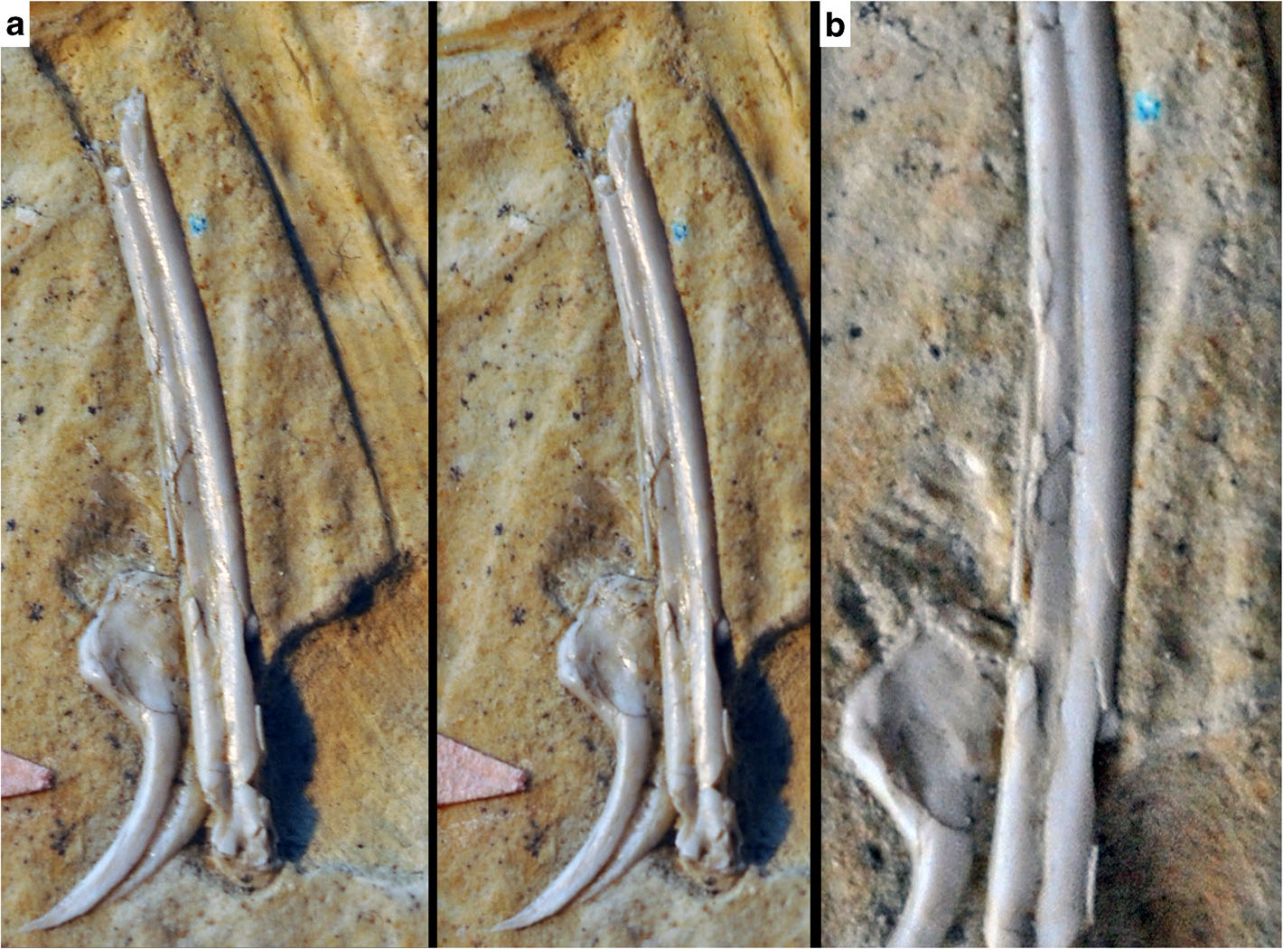
Details of manual phalanx I-1 of *Ostromia crassipes*, showing the regular development and non-collapsed margins of the longitudinal groove. **a**, stereophotograph. **b**, magnification of the shaft of the phalanx

**Fig. 5:**
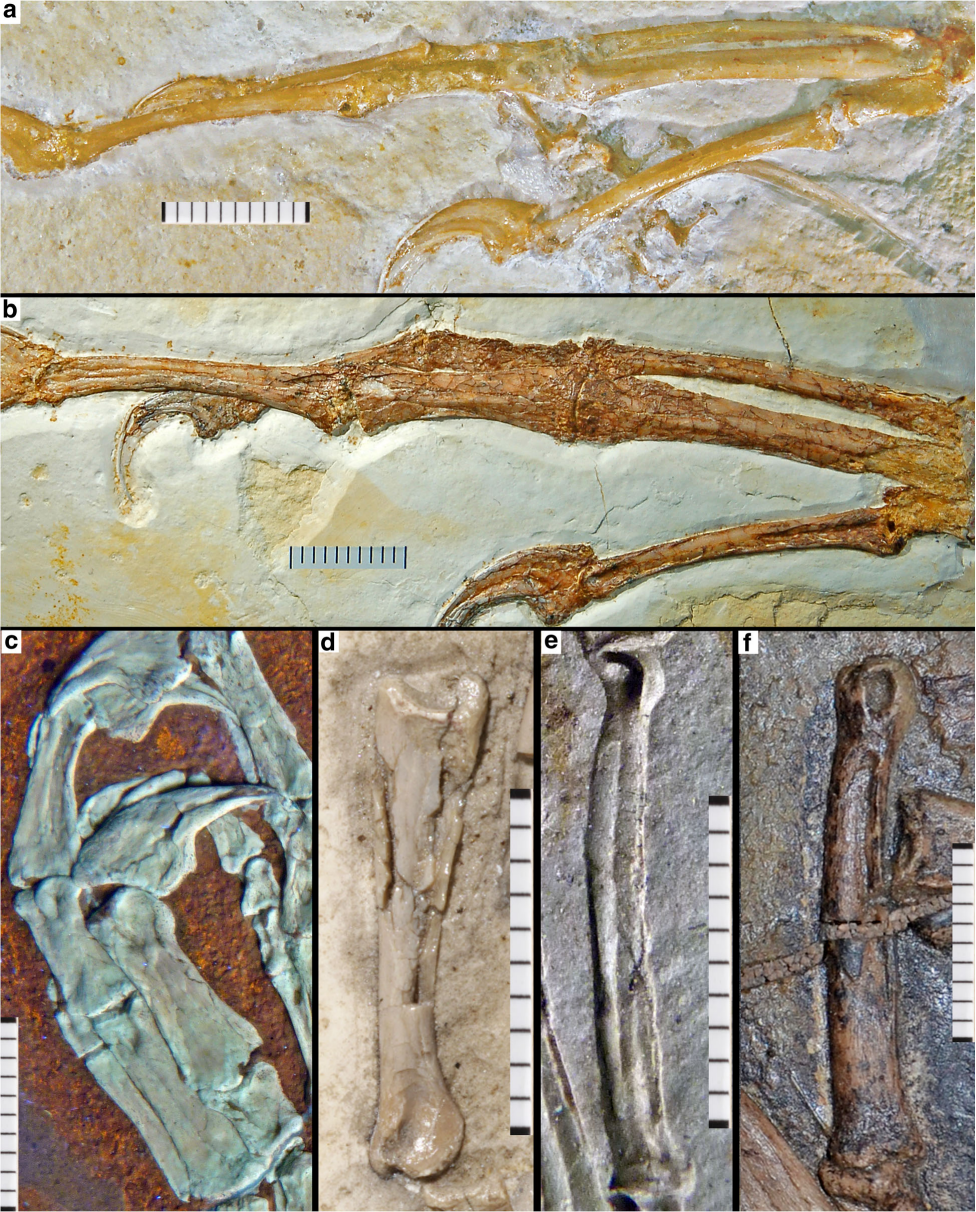
Manual phalanges of several other theropods preserved in highly compacted sediments, showing differences (**a-d, f**) and similarities (**e**) in preservation to the manual furrows in the Haarlem specimen. **a** Right manus of the Thermopolis specimen of *Archaeopteryx*, showing non-collapsed phalanges without furrows, as they are found in many specimens of this taxon (e.g. London, Berlin, Eichstätt, Munich, 11th specimen). **b** Right manus of the Solnhofen specimen of *Archaeopteryx*, showing partially collapsed and crushed phalanges with clearly broken margins. **c** Left manus of *Sciurumimus albersdoerferi* (BMMS BK 11) under UV light, showing partially compressed phalanges in this juvenile theropod. **d** Phalanx II-2 of *Compsognathus longipes* (SNSB-BSPG AS I 563), showing non-collapsed, but partially broken shaft. **e** Impression of phalanx I-1 of the holotype of *Anchiornis huxleyi* (IVPP V 14378), showing the impression of a regular longitudinal furrow, very similar to the impressions seen in the Haarlem specimen. **f** Phalanx I-1 of *Caudipteryx* (IVPP V 12430), showing collapse structure distally with clearly broken margins. Scale bars are in mm increments

Beside proportional differences, the Haarlem specimen shows a regular, well-developed longitudinal furrow on the exposed medial side of the preserved manual phalanx I-1 (Figs. 3b and 4). Although the margins of the phalanx show signs of compression, the furrow itself is intact and does not show any signs of breakage due to collapse of the bone (Figs. 3b and 4). Furthermore, the impression of the lateral side of phalanx III-3 shows a very similar, regular longitudinal ridge as counterpart to a furrow that was obviously present in this phalanx (Fig. 3b). The distal end of this phalanx is preserved as bone on the counterslab, and also shows the distal end of such a furrow towards the articular end of the medial side (Fig. 3b), indicating that these furrows were present on both sides of the manual phalanges; this is supported by the impression of this phalanx on the main slab. A longitudinal furrow is furthermore also present on the lateral side of a small preserved fragment of metacarpal III (Fig. 3a), again with no indication of being caused by collapse of this element, such as broken or irregular margins. None of the other specimens that can securely be identified as *Archaeopteryx* shows such regular furrows (e.g. Eichstätt, Munich, Thermopolis, and 11th specimens; CF and OR, pers. obs.; Fig. 5a), although collapse structures are present in various long bones of other specimens. In the Solnhofen specimen, for example, a longitudinal furrow seems to be present in the basal part of the right phalanx I-1 and II-2, but these grooves have irregular margins and show breaks at their deeper parts, indicating that they were caused by collapse of the hollow bone (OR, pers. obs.; Fig. 5b). Indeed, the absence or, when present, very typical morphology of such collapse structures in a wide variety of terrestrial vertebrates from the Solnhofen limestones, including lepidosaurs, crocoylomorphs, pterosaurs, and non-avialian theropods, and their inconsistent appearance in various elements or only parts of elements in other *Archaeopteryx* specimens differs drastically from the very regular structures found in all preserved manual phalanges in the Haarlem specimen, thus supporting our interpretation that the regular furrows in the manual elements are primary structures in the latter.

**Fig. 6:**
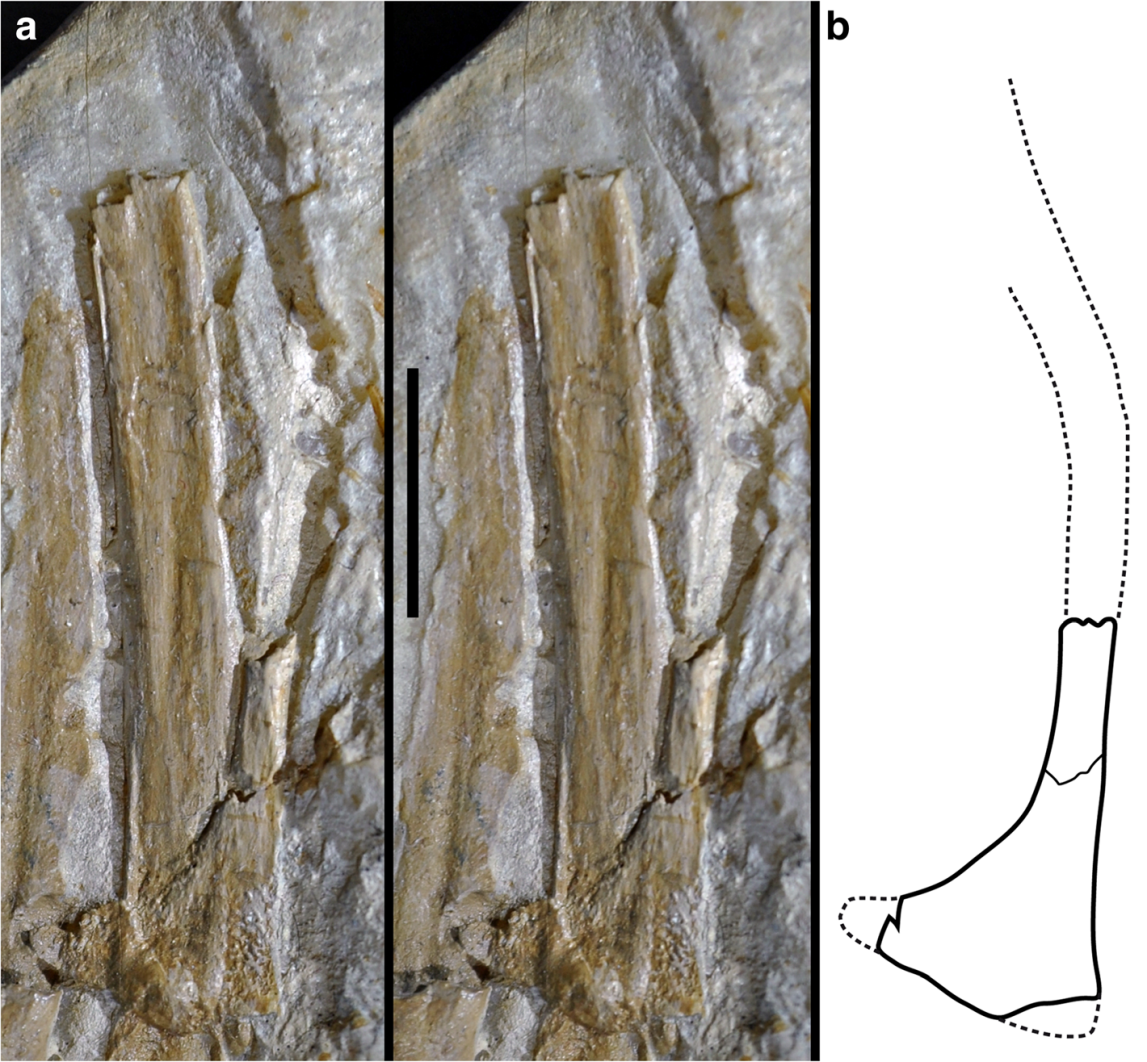
Pubic morphology of *Ostromia*. **a** Stereophotograph of the preserved pubis and impressions of the shaft, taken from a high quality cast at the BSPG. **b** outline reconstruction. Scale bar is 10 mm

**Fig. 7:**
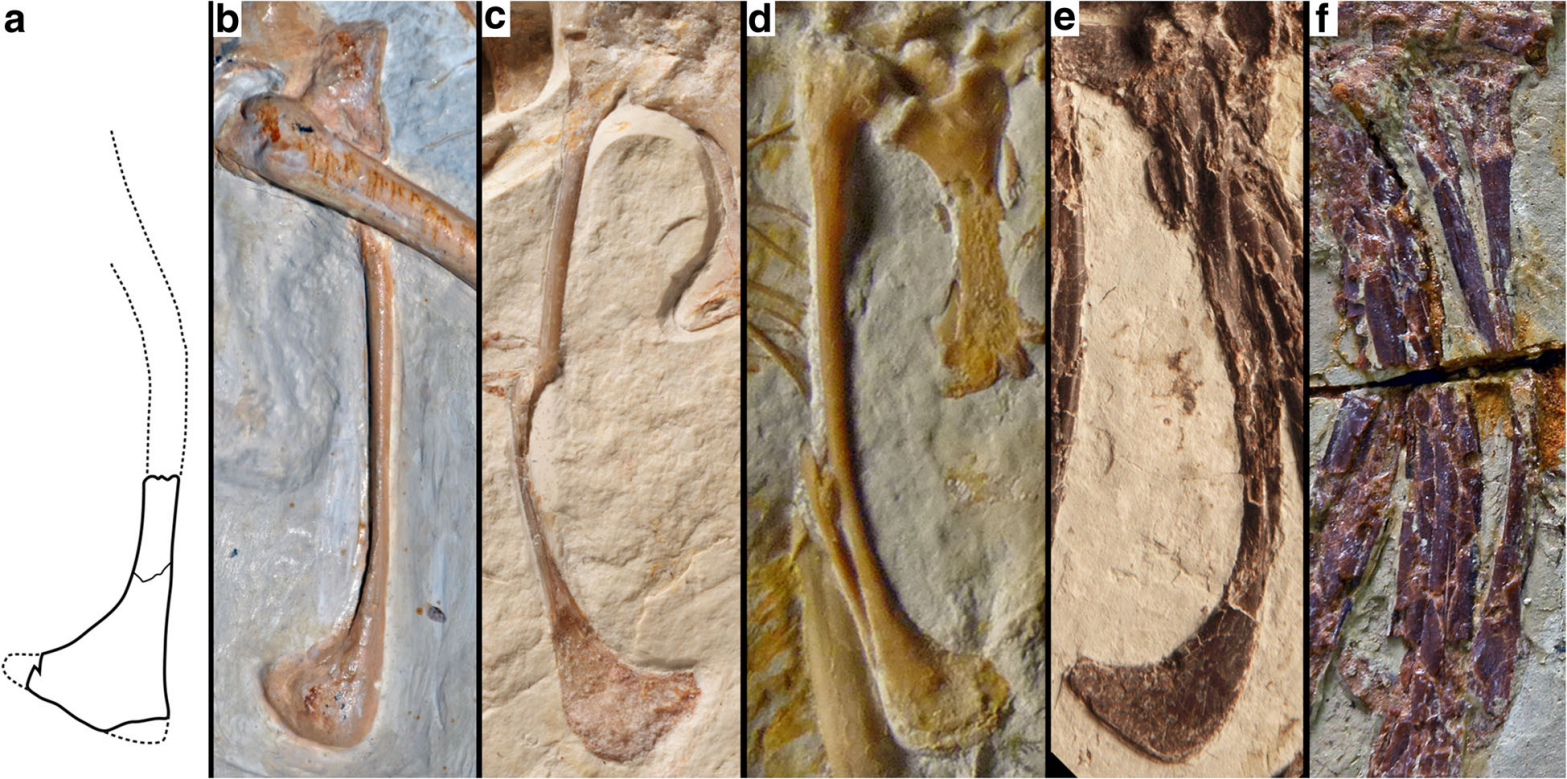
Comparison of pubic morphology of *Ostromia* (**a**) with *Archaeopteryx* (**b-d**) and *Anchiornis* (**e, f**). **a** Reconstructed pubis of *Ostromia crassipes*. **b** Pubis of the Berlin specimen of *Archaeopteryx* (photograph of high-quality cast at BSPG). **c** Pubes of the Munich specimen of *Archaeopteryx*. Note that the proximal shaft is of the left pubis, while the distal part is the right pubis, the proximal part of which is hidden by matrix. **d** Pubis of the 11th specimen of *Archaeopteryx*. Note that part of the shaft of the right pubis is visible distally, creating the impression of a flexed shaft. However, any impression of these structures would clearly show two pubic shafts, unlike the situation in the Haarlem specimen (see Fig. 5a). **e** Pubis of *Anchiornis* (BMNHC PH822; photo courtesy Rui Pei). **f** Pubis of *Anchiornis* (STM0-165; photo courtesy Wang Xiaoli)

Further differences can be found in the pubis, although only parts of the distal shaft and the pubic boot of the right pubis are preserved on the main slab of the Haarlem specimen, while the shaft of the left pubis is indicated by its impression (Fig. 6). Although the pubes were slightly compressed, and thus the shaft of the right pubis was slightly displaced anteriorly in respect to that of the left pubis (as e.g. in the Solnhofen and Thermopolis specimens), there is no indication of a break in the pubic shafts in the rather clear impression, and the exposure of the pubic boot in lateral view indicates that the pubis was embedded mainly transversely in the sediment. Thus, the preserved parts of the pubis and the impression clearly shows that the pubic shaft was strongly flexed, being anteriorly convex, in its medial part (Fig. 6). In those *Archaeopteryx* specimens, where the pubis is exposed in lateral view (i.e. Berlin, Solnhofen, Munich, 11th, and 12th specimen) the bone is slender with a straight to very slightly curved shaft (Fig. 7b-d). Only the Thermopolis specimen seems to show a slightly flexed pubic shaft [24], but the bone is seen in anterolateral view, making a final validation impossible. In contrast, the curving in the pubic shaft of the Eichstätt specimen is a preservational artefact, caused by the right pubic apron, which was displaced anteriorly during burial, forming a kink. However, the posterior margin of the pubic shaft is almost straight, as in the other *Archaeopteryx* specimens, and the slight apparent flexure of the pubis shafts in these specimens still differs considerably from the strongly flexed shafts in the Haarlem specimen. Furthermore, the pubic boot of the Haarlem specimen is triangular in outline, with an almost straight distal margin (Fig. 6), while in all other *Archaeopteryx* specimens, where the shape of structure can be verified, the pubic boot is distally convex and curves proximally posteriorly, resembling a the shape of a soup-ladle [9, 13] (Fig. 7b-d).

Having established significant differences between the Haarlem specimen and those specimens that can securely be referred to *Archaeopteryx*, the fragmentary nature of the former specimen make an identification of its affinities difficult. However, although the presence of longitudinal furrows on the manual phalanges has not been described in the literature, we found this character to be clearly present in *Anchiornis huxleyi*, based on own observations by one of us (CF) on the holotype of this species (Institute of Vertebrate Paleontology and Paleoanthropology IVPP V14378). Although the only preserved manus in this specimen is poorly preserved and has suffered from severe breakage and erosion of the bones, the longitudinal furrows are clearly visible on the impression of the manus on the counterslab (Fig. 3c, d). Despite generally poor bone preservation in the specimens from the Tiaojishan Formation, this observation could subsequently be confirmed in other specimens (STM0-52 [25]; XHPM 1084; CF pers. obs.). Imprints of metacarpal III in the right manus of STM0-52 indicates that the furrows were present on both sides of the bone, as it is the case in Haarlem specimen (see above). As in the case of the Haarlem specimen, the very regular appearance of these furrows in the specimens we examined (Fig. 5e) argues against an interpretation as simple collapse structures. Similar furrows also seem to be present in the specimens described by Pei et al. ([26]: figs. 19, 28-30, although from the photos provided it is impossible to determine with certainty whether these structures might result from collapse of the phalanges. Furthermore, such furrows also seem to be present in the manual phalanges of *Eosinopteryx* [27], but they are clearly absent in *Xiaotingia* [4]. Outside Avialae, a longitudinal furrow can be found on phalanx I-1 (= phalanx II-1 based on the terminology of Xu et al. [28]) of the basal troodontid *Jianianhualong tengi* (DLXH 1218 [28]). Less well-developed furrows are also present in at least some manual phalanges of the basal tyrannosauroids *Guanlong* (IVPP V14531; OR, pers. obs.) and *Yutyrannus* (ZCDM V5001 [29]), and the paravians *Sinornithoides* (IVPP V9612; OR, pers. obs.) and *Sinornithosaurus* (IVPP V12811 [30]). A specimen of *Caudipteryx* (IVPP V12430; CF, pers. obs.) shows a weakly developed furrow on phalanx II-2 only, but it cannot be completely excluded that this structure is due to collapse. However, these furrows are less well-developed than in the Haarlem specimen and do not occur on all manual phalanges or the metacarpals (as with the presence of a groove on one phalanx only in *Jianianhualong*).

In addition, the Haarlem specimen resembles *Anchiornis* with respect to its pubis morphology. Although not preserved in the holotype [31], the specimens described by Hu et al. [32], specimen BMNHC PH822 described by Pei et al. ([26]: fig. 32), and specimens STM0-165, STM0-52 [25] and STM-0-118 [33] show that the distal two thirds of the pubic shafts in this taxon are strongly flexed, and that the pubic boot has a similar, triangular outline (Fig. 7e, f).

### Phylogenetic and taxonomic status of the Haarlem specimen

The parsimony analysis retained more than 15.580 equally parsimonious trees with a length of 2624 steps. The strict consensus tree shows a reasonably good resolution and largely conforms to the tree published by Foth et al. [9] (see Additional file 1). The Haarlem specimen did not cluster with *Archaeopteryx*, but was found in a polytomy together with *Pedopenna*, *Eosinopteryx*, and *Anchiornis*, to the exclusion of *Xiaotingia*, which represents the sister taxon to this polytomy (Fig. 9). The bootstrap values of the phylogeny are generally low, and only two additional steps are needed to move the specimen as sister taxon to *Archaeopteryx*. However, as only 46 characters could be coded for the Haarlem specimen, and most of these are plesiomorphies at the node of Avialae, two additional steps actually represent a rather large part of the character transformations that help placing this taxon.

Apart from the fact that there are no anatomical characters that unambiguously support a referral to *Archaeopteryx*, the results of the phylogenetic analysis thus support the removal of the Haarlem specimen from this genus. Given the very incomplete and rather poor preservation of the specimen, the question arises whether to base a new taxon on this material. However, a species name for this specimen already exists, as it was originally named as a new species of the pterosaur *Pterodactylus*, *P. crassipes*, by von Meyer [11]. Thus, in order to conserve this name, and as the specimen cannot be referred to any known genus, we propose a new generic name for *P. crassipes*.

Like our analysis, previous studies have found *Anchiornis* to form a clade with *Xiaotingia* (e.g [4, 8–10, 33, 34]), but also *Eosinopteryx* [10, 26]. As outlined above, the available character evidence also place the Haarlem specimen in this lineage. Based on this topology, and given that this clade represents an important taxon within the plexus of derived maniraptorans surrounding the evolutionary origin of avialans, we propose a new clade, named Anchiornithidae.

**Systematic Palaeontology**

Theropoda Marsh, 1881 [35]

Maniraptora Gauthier, 1986 [36]

Anchiornithidae tax. Nov.

*Type genus. Anchiornis* Xu, Zhao, Norell, Sullivan, Hone, Erickson, Wang, Han, and Gao, 2009 [31].

*Definition.* Anchiornithidae is a stem-based taxon defined as all maniraptoran theropods that are more closely related to *Anchiornis huxleyi* than to *Passer domesticus*, *Archaeopteryx lithographica*, *Dromaeosaurus albertensis*, *Troodon formosus*, or *Oviraptor philoceratops*.

*Diagnosis.* Based on the evaluation of character transformations in the phylogenetic hypothesis presented here, the following morphological characters can currently be used to diagnose Anchiornithidae: Nutrient foramina on dentary placed in deep groove (convergently present in most troodontids and some other coelurosaurs); anterior dentary teeth smaller, more numerous, and more closely appressed than those in the middle of the tooth row (convergently present in most troodontids); anterior edge of acromion margin of scapula laterally everted or hooked (convergently present in several oviraptorosaurs and more derived avialans); medial surface of proximal fibula flat (convergently present in alvarezsaurids, therizinosauroids and derived avialans); fan-shaped posterior dorsal neural spines (convergently present in compsognathids and some derived avialans); extensive large pennaceous feathers on metatarsus and pes (convergently present in *Microraptor* and *Sapeornis*).

Further possible diagnostic characters include the following: Retroarticular process of the mandible curves posterodorsally (only known in *Xiaotingia*; convergently present in derived ornithomimosaurs and aves); shallow Meckelian groove in dentary (only known in *Xiaotingia*; convergently present in several tyrannosauroids and derived avialans); presence of a posterior flange on manual phalanx II-1 (only known in *Anchiornis*; convergently present in some dromaeosaurids and advanced avialans); presence of a shelf-like supraacetabular crest of ilium (currently only known in *Anchiornis*; reversal to the basal tetanuran condition, convergently present in alvarezsaurids and several avialans); anteriorly convex pubic shafts (present in *Anchiornis* and the Haarlem specimen, apparently absent in *Eosinopteryx*; convergently present in some dromaeosaurids and more derived avialans).

*Ostromia* gen. nov.

*Etymology.* The generic name honours the late John Ostrom, who identified the Haarlem specimen as a theropod.

*Ostromia crassipes* von Meyer, 1857 [11]

*Holotype.* Teylers Museum TM 6928, 6929, part and counterpart of a fragmentary skeleton.

*Locality and horizon.* Jachenhausen locality, near Riedenburg, Bavaria, Germany. Early Tithonian laminated limestones of the Painten Formation [37].

*Diagnosis.* Due to the poor preservation of the holotype, this species cannot be distinguished from other theropods strictly on apomorphic characters; only a differential diagnosis can be given. *Ostromia crassipes* differs from most theropods with the probable exception of *Anchiornis* and *Eosinopteryx* (as closest relatives) in the presence of longitudinal furrows on both sides of all preserved manual phalanges and at least metacarpal III. The taxon differs from other anchiornithids in an unusually small first manual ungual and other proportions. Measurements for an almost complete specimen of *Anchiornis* were given by Hu et al. [32], and this specimen differs from *Ostromia* in the relative length of metacarpal III in comparison to metacarpal I (2.46 versus 2.21), the length of the first manual ungual in comparison to metacarpal I (1.26 versus 0.9), and the length of the tibiotarsus in comparison to the metatarsus (1.93 versus 1.67) (Table 2). In contrast, only a few selected measurements were given for *Eosinopteryx* [27], so that the only comparable ratio is that between the tibiotarsus and the metatarsus, which is 1.96 in *Eosinopteryx*, as opposed to 1.67 in *Ostromia*. Furthermore, although no measurements are given, the photo of the manus of *Eosinopteryx* clearly shows that manual ungual I is longer than metacarpal I in this taxon, rather than shorter, as it is the case in *Ostromia*.

Apart from the geographic and stratigraphic differences (*Anchiornis* and *Eosinopteryx* are of Oxfordian age, whereas *Ostromia* comes from the early Tithonian; Schweigert, pers. com.), these differences indicate that the European anchiornithid is different from its Chinese cousins so that the proposal of a new generic name seems justified. A full anatomical description of this specimen was provided by Ostrom [16].

## Discussion

### *Ostromia* as a separate genus

In his detailed description of the Haarlem specimen, Ostrom [16] noted several possible differences in proportion between this and other specimens of *Archaeopteryx* then known, most notably in the relative length of the metatarsus and the manual unguals. However, he assigned these differences either to preservational artifacts (in the case of the metatarsus) or individual variation. The re-exanimation of the Haarlem specimen in combination with a broad-scale phylogenetic analysis did not find any shared unique characters of this specimen with the genus *Archaeopteryx*, but highlighted several proportional and anatomical differences and thus strongly implies that this specimen is not an *Archaeopteryx*, but represents a different genus and species, *Ostromia crassipes*, which is closely related to the basal avialan *Anchiornis* from the lower Upper Jurassic of China.

Although the differences in proportions are statistically significant, it must be noted that the statistical power of the statistical tests performed is low, given the very low sample size. However, these results support the notion that the specimen is significantly different from *Archaeopteryx*. More importantly, in the absence of any discrete anatomical characters that would support a referral of the Haarlem specimen to *Archaeopteryx*, and the general anatomical similarity of many basal paravian theropods, these differences in basically all of the few comparable proportions are a strong indication that this specimen should be removed from that genus.

The nature of the osteological features that distinguish the Haarlem specimen from *Archaeopteryx* could be questioned on the basis of the incompleteness of the specimen and strong taphonomic compaction. Although the reconstruction of the pubis, for instance, is partly based on imprints in the matrix, we interpret the shape to be reliable, based on the general quality of the imprints, as seen in other parts of the skeleton, such as the manual and pedal phalanges. Furthermore, as the preserved remains indicate that the specimen was embedded lying on its side, there is no reason to assume that compression would affect the anteroposterior flexure of the pubic shafts and the shape of the pubic boot, both of which are preserved in lateral view.

As noted above, the phalanges themselves show no signs of collapse due to compression, so that the longitudinal furrows are interpreted to be real structures. As with most long bones of coelurosaurian theropods, manual phalanges are usually thin-walled and hollow. Especially in specimens from strongly compressed sediments, such as the lithographic limestones of southern Germany and Las Hoyas (Spain) or the lacustrine deposits of the Daohugou and Jehol beds, this might lead to a collapse of these bones, resulting in often crushed elements that might show unnatural depressions or indentations (see e.g. Fig. 5b–f). As noted above, however, the collapse structures in the phalanges and other long bones of other *Archaeopteryx* specimens from the laminated limestones of southern Germany differ considerably from the very regular furrows found in the Haarlem specimen (Figs. 4 and 5). Various basal birds from the Jehol group show a combination of longitudinal furrows and multiple, irregular breakages of phalanges and metacarpals, while in many other specimens these bones stay intact (extensively figured in Chiappe and Meng [38]). Although the holotype and various referred specimens of *Anchiornis* show severe cases of breakage and erosion in the skeleton, including the forelimbs, we interpret the repeatable presence of longitudinal furrows along the main axis in the manual phalanges in multiple specimens of *Anchiornis* to be a real structure and not an artefact of compression. This is supported by our own observations of several specimens, including the holotype (IVPP V14378), in which these furrows (or their natural molds) are more regular than would be expected for collapse structures (Figs. 3c, d and 5e).

### Theropods in the Solnhofen archipelago

**Fig. 8:**
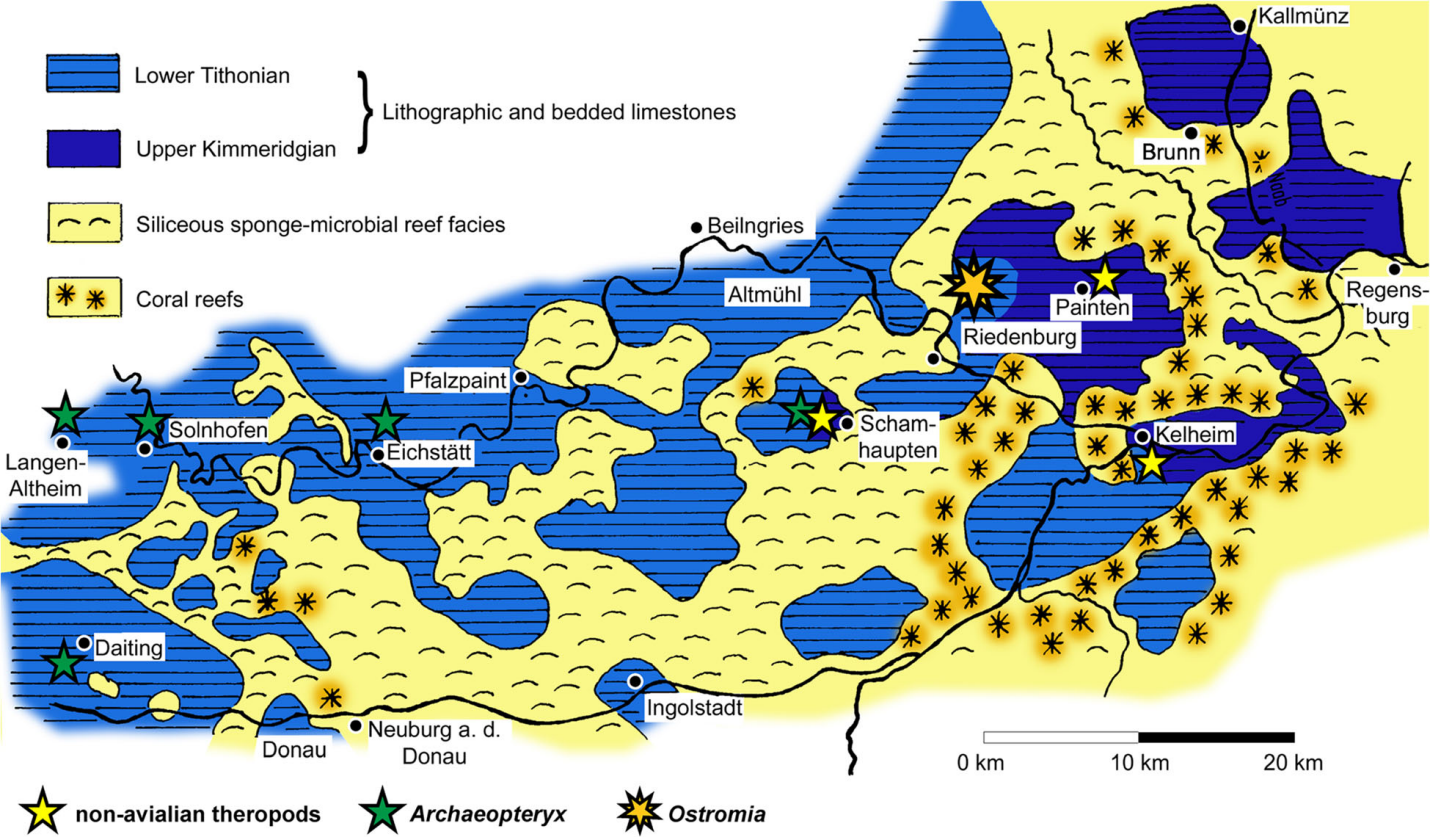
Occurrence of *Ostromia* within the “Solnhofen Archipelago” and distribution of theropod dinosaurs in the Jurassic of that area. Modified from Butzmann et al. [72]

The result of our re-evaluation of the Haarlem specimen has far-reaching consequences for our understanding of the theropod fauna of the Solnhofen Archipelago, but also for the early evolution and biogeography of maniraptoran theropods in general. All described skeletal specimens of *Archaeopteryx* come from the Solnhofen, Eichstätt and Daiting area [13], with the exception of the so far undescribed 12th specimen, which comes from Schamhaupten [39], some 30 km to the east (Fig. 8). *Ostromia* comes from the locality of Jachenhausen [13], 10 km further to the north-east from Schamhaupten. The Jachenhausen locality is at the westernmost rim of the Painten Basin, one of the easternmost plattenkalk basins within the Solnhofen archipelago. These eastern basins (Schamhaupten, Painten, Hienheim, Kelheim, and Brunn basins) seem to have a higher terrestrial influence than the western basins, as indicated by abundant plant remains, lepidosaurs, and the non-avian theropod dinosaurs *Compsognathus*, *Juravenator*, and *Sciurumimus* [40–43] (Fig. 8). The identification of *Ostromia* as an anchiornithid adds to this remarkable diversity of theropods in these eastern areas, and underlines the faunal differences with the western occurrences, for which *Archaeopteryx* remains the only theropod recorded, although in greater abundance than the different taxa in the eastern basins. Apart from a slight stratigraphic difference between some of the eastern and western plattenkalks, this most likely reflects different settings in these areas: whereas terrestrial organisms in the eastern area might have come directly from the larger land mass of the Bohemian massif to the east or from islands within the directly adjacent coral reef zone [44], the western occurrences were considerably more remote from any larger land mass, and terrestrial animals might only come from isolated small islands [42]. In this setting, even very limited flight capabilities might have represented a crucial advantage for a taxon like *Archaeopteryx* to invade these more remote habitats. Thus, the occurrence of this taxon in a more marine setting represents indirect further evidence for at least some sort of flight capabilities in *Archaeopteryx*.

### The radiation and dispersal of maniraptoran theropods

**Fig. 9:**
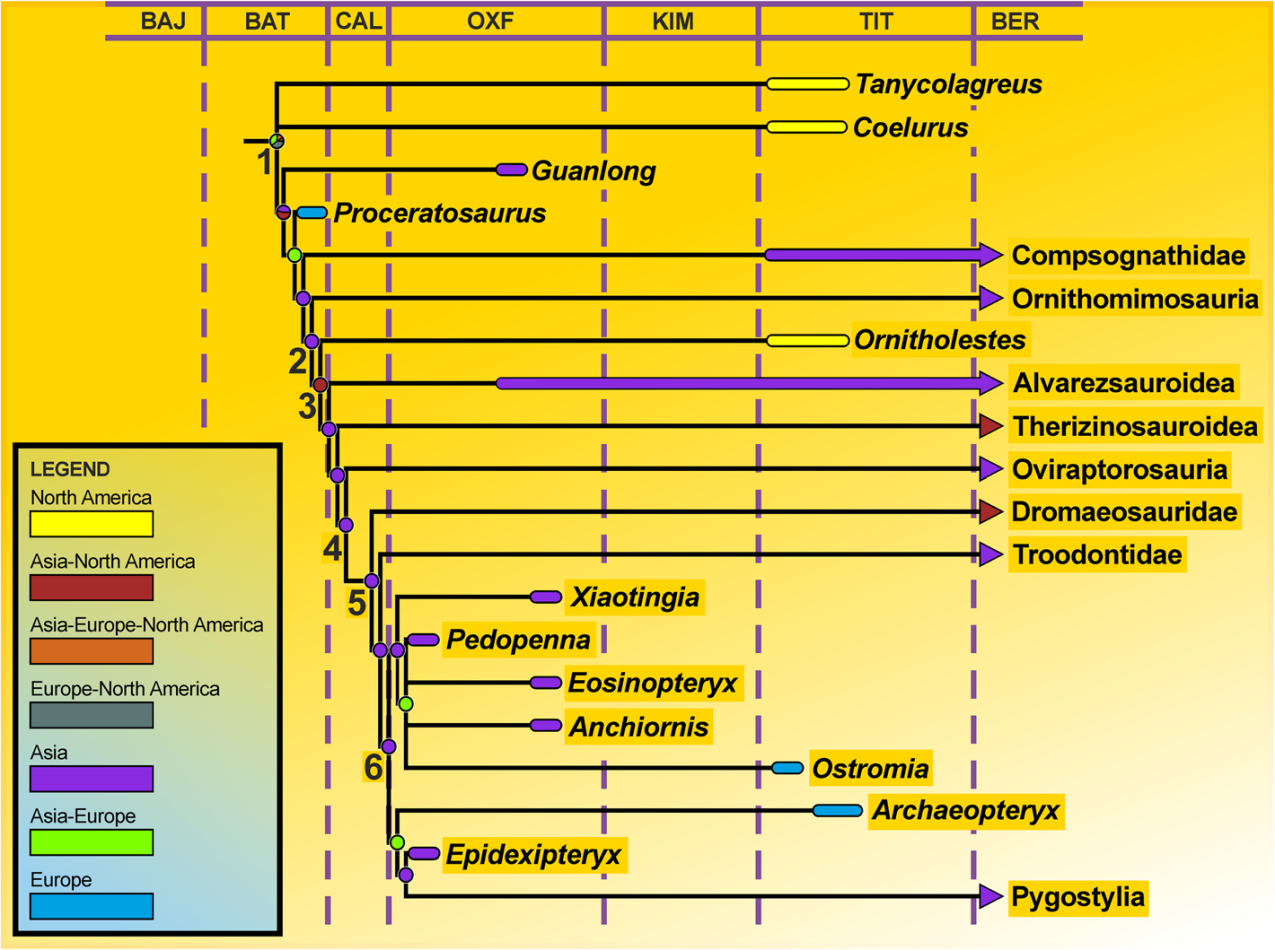
Time calibrated simplified phylogeny of maniraptoran theropods, indicating place of origin of the different clades. The relationships and stratigraphic and geographic distribution of maniraptorans indicate a rapid radiation in the late Middle to earliest Late Jurassic in eastern Asia. Node labels and abbreviations: 1, Coelurosauria; 2, Maniraptoriformes; 3, Maniraptora; 4, Pennaraptora; 5, Paraves; 6, Avialae; BAJ, Bajocian; BAT, Bathonian; BER, Berriasian; CAL, Callovian; KIM, Kimmeridgian; OXF, Oxfordian; TIT, Tithonian. Based on a phylogenetic analysis of 2 outgroups and 130 coelurosaurian ingroup taxa and 561 characters and the results of a S-DIVA analysis

Apart from its significance for our understanding of the fauna of the Solnhofen archipelago, the identification of *Ostromia* as an anchiornithid represents an enormous range extension for this paravian clade from the Tiaojishan Formtaion of China to central Europe. All three S-DIVA analyses (with the entire data set and the two time-sliced sets) indicate that, whereas there is no clear biogeographic signal for the origin and initial diversification of coelurosaurs, the radiation of maniraptoran theropods more derived than the basal taxon *Ornitholestes*, including Alvarezsauroidea, Oviraptorosauria, Paraves/Eumaniraptora, Troodontidae, and Avialae, most likely happened in eastern Asia, as all nodes leading to the major clades within Maniraptora are optimized for this area (Fig. 9; see Additional file 1). Alternative phylogenetic positions like that for the aberrant theropod *Epidexipteryx* (basal Avialae ([45, 46]; this study) vs. basal Paraves [5, 27, 34, 47] vs. basal Oviraptorosauria [8, 10] or *Anchiornis* (basal Deinonychosauria [4, 34] vs. basal Troodontidae [32, 47] vs. basal Avialae ([8, 31], this study) would not change the outcome of the analyses.

In this scenario, the occurrences of an anchiornithid and *Archaeopteryx* in the Solnhofen Archipelago represent two phylogenetically (although not necessarily temporally) distinct dispersal events from eastern Asia to central Europe in the Late Jurassic. Given the probably Oxfordian age of the anchiornithids from the Tiaojishan Formation [6], basically all maniraptoran clades must have been established by this time, including the lineage leading to *Archaeopteryx* and higher avialans [48]. On the other hand, the oldest certain records of coelurosaurs are Bathonian in age and come from Europe [49] and central Asia [50]. As eastern Asia seems to have been isolated from the rest of Laurasia by epicontinental seas by the Callovian at the latest [51], the biogeographic pattern, together with the stratigraphic occurrences of taxa and palaeogeographic reconstructions indicate a rapid, seemingly explosive radiation of maniraptoran theropods in isolation in eastern Asia in the late Middle Jurassic (Bathonian-Callovian), with a subsequent dispersal from eastern Asia towards Europe and North America, in the Late Jurassic (Fig. 9), and further radiations and dispersals to South America [52, 53], Madagascar [54] and Africa [55] during the Cretaceous. This scenario is supported by another recent analysis of dinosaurian biogeography [56], which found a peak in connectivity between the continents in the Late Jurassic, indicating that Europe was a “turntable” for dinosaur dispersal during that time.

Although this rapid, eastern Asian radiation of advanced maniraptorans is our preferred hypothesis in the light of the currently available evidence, it should be noted that it cannot be excluded that this might, at least partially, be an artifact of the fossil record. Prior to the recent description of abundant maniraptoran theropods from the Oxfordian of China ([4, 5, 27, 31, 34, 45, 46, 57, 58]), no certain pre-Kimmeridgian representatives of this clade were known. Late Middle Jurassic small theropod remains are exceedingly rare globally, but a few specimens, mainly isolated teeth, have been referred to paravian clades, most notably dromaeosaurids (e.g. [59–61]). However, teeth similar to those found in dromaeosaurids are also present in basal tyrannosauroids ([49]) and, probably, the juvenile dentition of more basal theropods ([40]), so more conclusive remains would be needed to prove the existence of pre-Late Jurassic paravians in areas outside eastern Asia. Likewise, most possible maniraptoran remains from Late Jurassic extra-Asian localities are fragmentary (e.g. [62–64]) and rather inconclusive. Even if confirmed, the presence of maniraptoran theropods in the Kimmeridgian-Tithonian of Europe or the Morrison Formation would not contradict the hypothesis presented here, as they might result from a rapid Late Jurassic dispersal of this group over the Laurasian continents.

However this may be, the biogeographic pattern presented above might furthermore be mirrored by goniopholid crocodiles [65] and some pterosaur clades [66], which also seem to have expanded their range from eastern Asia towards Europe in the Late Jurassic. Palaeogeographic reconstructions indicate that at least a narrow marine barrier, probably with numerous islands, existed between Asia and Europe up to the Tithonian, when these landmasses might have been joined [67]. Whereas for crocodiles, different tolerances to brackish or even marine waters were evoked to explain the ability to cross the still remaining marine barrier through island hopping [65], flight ability was probably the key factor in the biogeographical evolution of pterosaurs [66]. For maniraptorans, small body size of the taxa at the base of all major lineages [68] might have made oversea dispersal by rafting possible [69], thus facilitating the crossing of the epicontinental barriers between Asia and Europe.

The separate Asian origin of the lineages of *Ostromia* and *Archaeopteryx* implied by the biogeographic analysis indicates that this dispersal might have happened in several independent waves, as has recently been suggested for the evolution of oscine passerines in the Cenozoic [70]. As exemplified by *Archaeopteryx*, flight ability might have further increased the dispersal potential for paravians, in which some form of flight capacity most probably evolved more than once [9, 34].

## Conclusions

A re-evaluation of one of the twelve skeletal specimens referred to the ‘Urvogel’ *Archaeopteryx*, the Haarlem specimen, revealed that this specimen represents a separate taxon, *Ostromia crassipes*. Phylogenetic analysis identifies *Ostromia* as the first representative of the basal avialian clade Anchiornithidae outside eastern Asia. In combination with a biogeographic analysis, a rapid radiation of maniraptoran theropods in eastern Asia with a subsequent dispersal of many lineages in the Late Jurassic is indicated; dispersal of maniraptorans was facilitated by small body size of basal members of all clades and, possibly, several independent acquisitions of flight capabilities. In the fragmenting world of Pangean break-up during the Late Jurassic and Cretaceous, increased dispersal potential might have been a key factor to explain the success of maniraptoran, and especially avialian theropods, with dispersal events being followed by endemic radiations of different clades [71].

## Additional file

Additional file 1: Details on phylogenetic and biogeographic analyses. (PDF 5013 kb)

## Institutional abbreviations

BMMS: Bürgermeister Müller Museum, Solnhofen, Germany
BMNHC: Beijing Museum of Natural History, Beijing, China
DLXH: Dalian Xinghai Museum, China
IVPP: Institute of Vertebrate Paleontology and Paleoanthropology, Beijing, China
SNSB-BSPG: Staatliche Naturwissenschaftlichen Sammlungen Bayerns-Bayerische Staatssammlung für Paläontologie und Geologie, Munich, Germany
STM: Shandong Tianyu Museum of Nature, China
TM: Teylers Museum, Haarlem, the Netherlands
XHPM: Xinghai Paleontological Museum of Dalian, China
ZCDM: Zhucheng Dinosaur Museum, Shandong, China
