# Supplementary material for: Re-evaluation of the Haarlem Archaeopteryx and the radiation of maniraptoran theropod dinosaurs
Source: BMC Evol Biol. 2017 Dec 2;17:236. doi: 10.1186/s12862-017-1076-y (PMC5712154; doi:10.1186/s12862-017-1076-y)
Supplement: Additional file 1: — Details on phylogenetic and biogeographic analyses. (PDF 5013 kb) [file 12862_2017_1076_MOESM1_ESM.pdf]

Supplementary Materials for

Re-evaluation of the Haarlem *Archaeopteryx* and the radiation of maniraptoran

Christian Foth, Oliver W. M. Rauhut*

correspondence to: o.rauhut@lrz.uni-muenchen.de

1. Additional information on the phylogenetic analysis

For the phylogenetic analysis we used an updated version of the matrix of Foth et al. [S1]. Several character codings were revised, and one additional character was added. Thus, all codings for character 145 [Flexor tubercles of manual unguals proximal (0) or displaced distally from articular end (1) or proximodistally elongated with proximal end close to articular facet (2)] were revised, as many coelurosaurs, including *Archaeopteryx* and *Ostromia*, actually show a distal displacement of the flexor tubercle (state 1), whereas originally mainly ornithomimosaurs were coded with this character state. The coding for character 172 [Pubic shaft straight (0) or distal end curves anteriorly, anterior surface of shaft concave in lateral view (1) or anterior surface of shaft convex in lateral view (2)] was changed from 0 to 2 for *Anchiornis*, as this taxon clearly shows a flexed pubic shaft (see main text), and from 0 to ? for *Xiaotingia*, as only the proximalmost part of the shaft is preserved in this taxon [S2]. Character 284 was slightly modified to include also ungual III of the manus, as this usually shows the same morphology as ungual II, and thus to make this character codable for taxa in which the ungual of the second digit might note be preserved, as in *Ostromia*:

284. With proximal articular surface of ungual orientated vertically, dorsal surfaces of manual ungual II and III do not (0) or do arch higher than level of dorsal extremity of proximal articular surface (1).

The new character concerns the presence of the longitudinal furrows on the manual phalanges:

Character 561: Longitudinal furrows on medial and lateral side of manual non-ungual phalanges: absent (0), present (1).

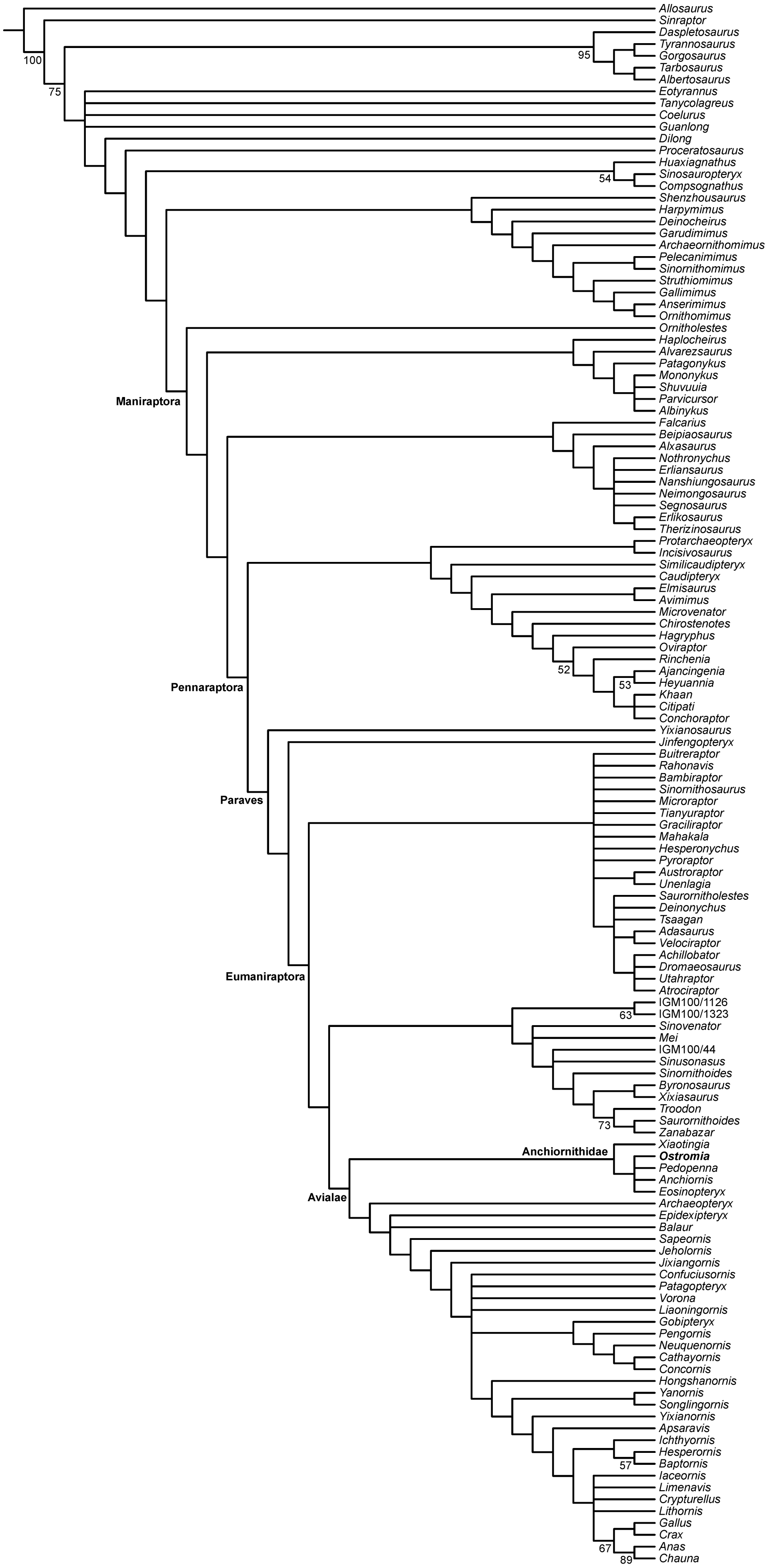

Fig. S1. Strict consensus tree resulting from the phylogenetic analysis, showing the phylogenetic position of *Ostromia crassipes*.

2. Results of the biogeographic analyses

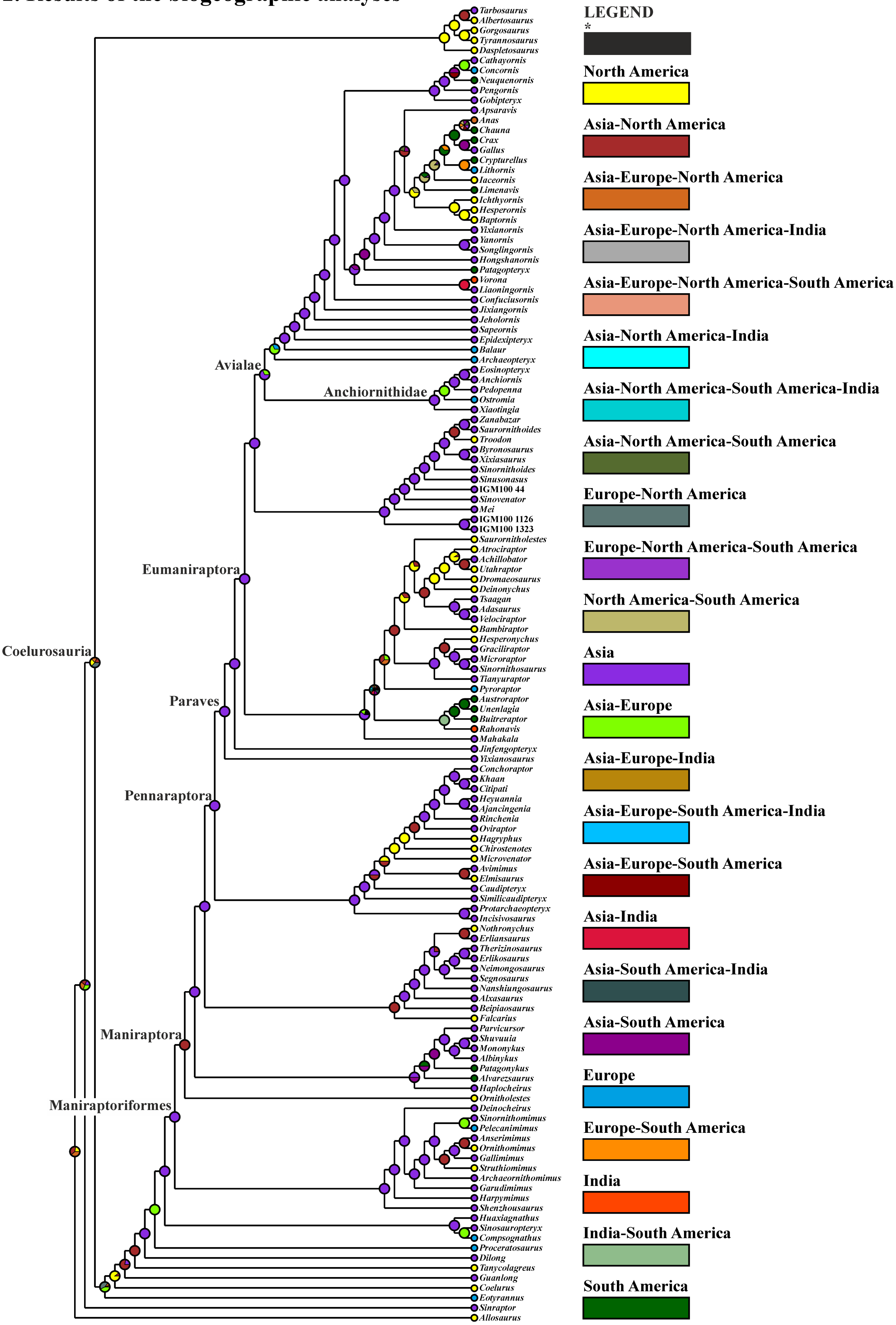

Fig. S2. Results of the S-DIVA analysis of coelurosaur biogeography, including all taxa.

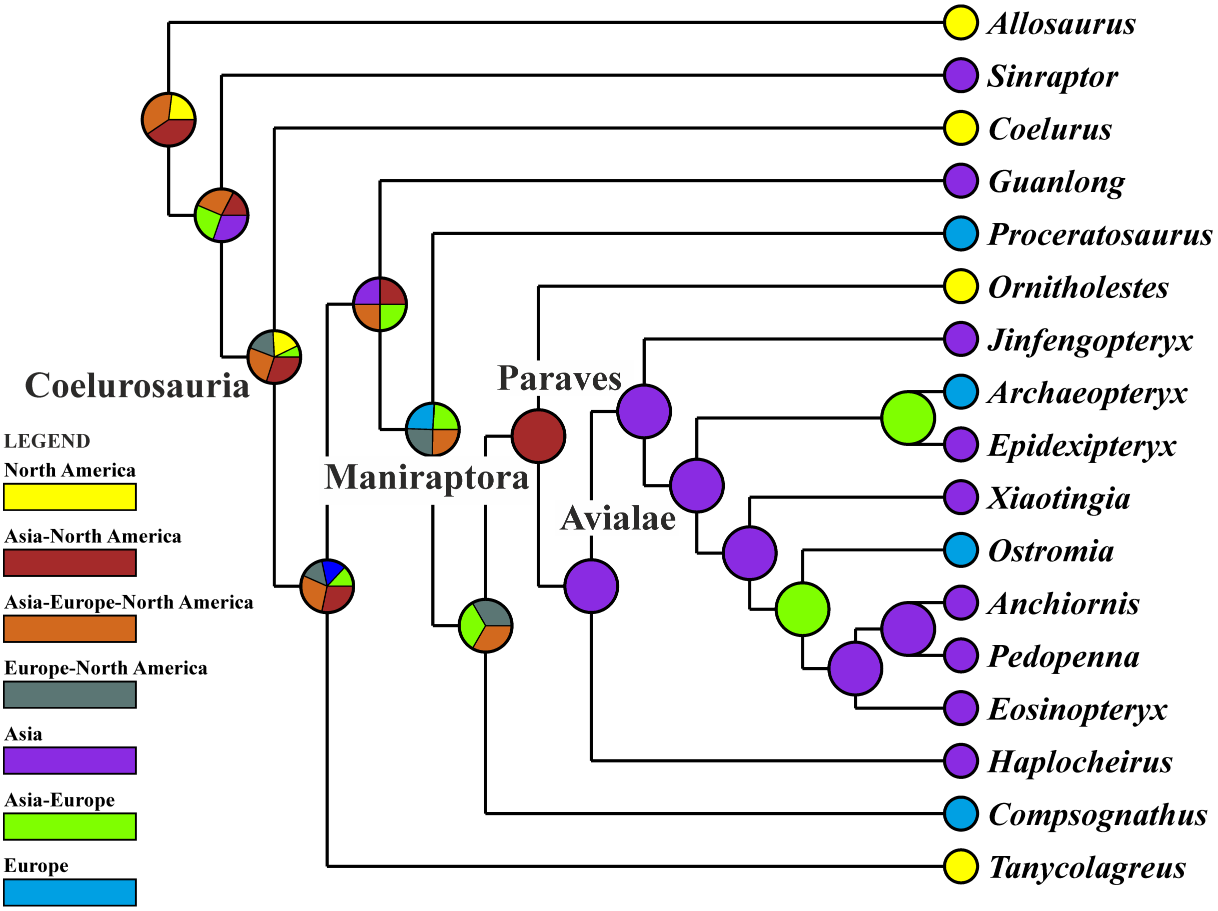

Fig. S3. Results of the time-sliced S-DIVA analysis, including only Jurassic taxa.

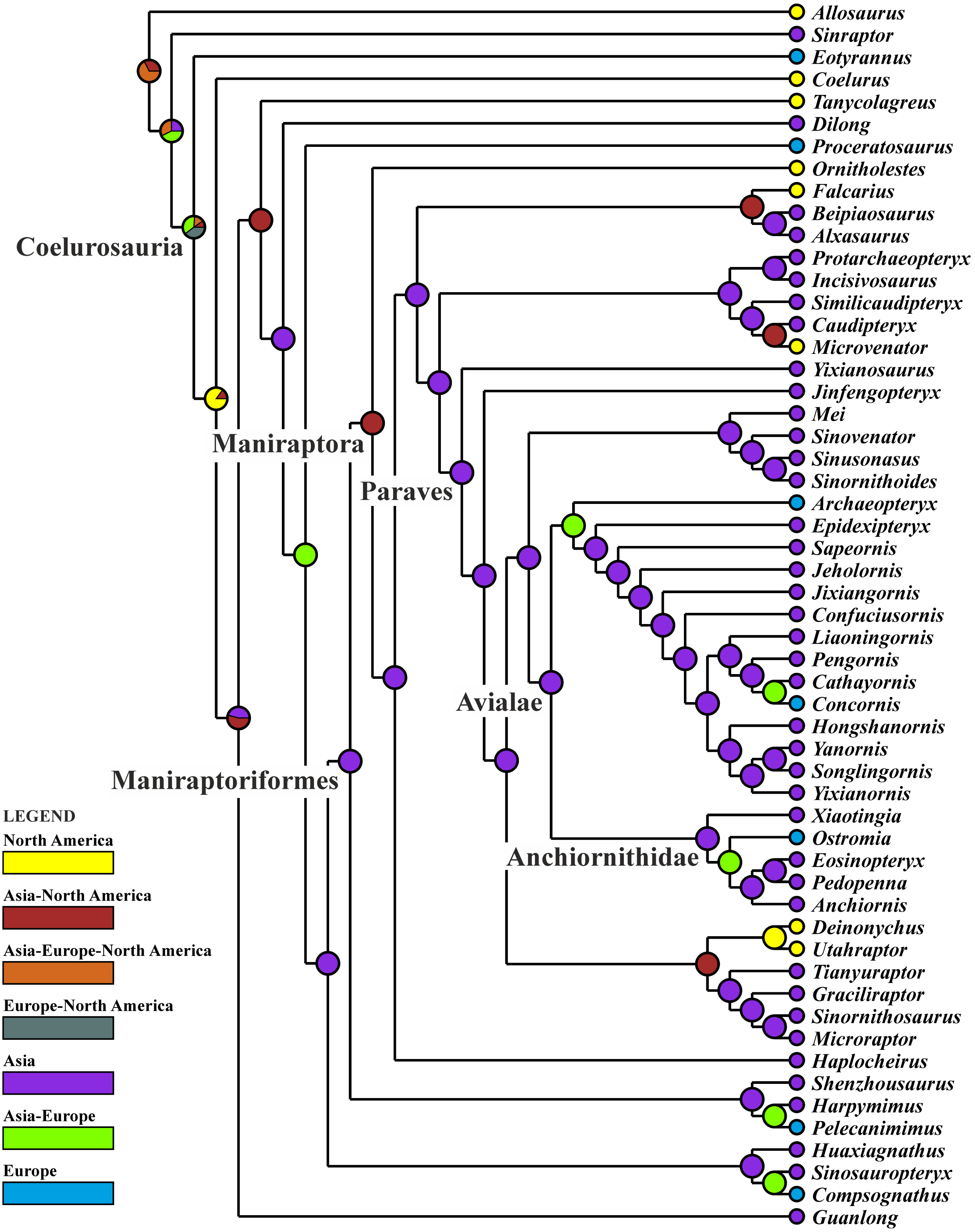

Fig. S4. Results of the time-sliced S-DIVA analysis, including Jurassic and Early Cretaceous taxa.

References

S1. Foth C, Tischlinger H, Rauhut OWM. New specimen of *Archaeopteryx* provides insights into the evolution of pennaceous feathers. Nature. 2014;511:79-82.

S2. Xu X, You H-L, Du K, Han F. An *Archaeopteryx*-like theropod from China and the origin of Avialae. Nature. 2011;475:465-70.
